# A joint model of longitudinal pharmacokinetic and time-to-event data to study exposure–response relationships: a proof-of-concept study with alectinib

**DOI:** 10.1007/s00280-024-04698-w

**Published:** 2024-07-11

**Authors:** Lishi Lin, Vincent van der Noort, Neeltje Steeghs, Gerrina Ruiter, Jos H. Beijnen, Alwin D. R. Huitema

**Affiliations:** 1 Department of Pharmacy & Pharmacology, The Netherlands Cancer Institute-Antoni van Leeuwenhoek Hospital, Plesmanlaan 121, 1066 CX Amsterdam, The Netherlands; 2 Department of Biometrics, The Netherlands Cancer Institute-Antoni van Leeuwenhoek Hospital, Amsterdam, The Netherlands; 3 Department of Medical Oncology, The Netherlands Cancer Institute-Antoni van Leeuwenhoek Hospital, Amsterdam, The Netherlands; 4 Department of Thoracic Oncology, The Netherlands Cancer Institute-Antoni van Leeuwenhoek Hospital, Amsterdam, The Netherlands; 5 Department of Pharmaceutical Sciences, Utrecht University, Utrecht, The Netherlands; 6 Department of Pharmacology, Princess Máxima Center for Pediatric Oncology, Utrecht, The Netherlands; 7 Department of Clinical Pharmacy, University Medical Center Utrecht, Utrecht University, Utrecht, The Netherlands

**Keywords:** Exposure response, Mixed effects models, Survival analysis, Alectinib

## Abstract

**Purpose:**

In exposure–response analyses of oral targeted anticancer agents, longitudinal plasma trough concentrations are often aggregated into a single value even though plasma trough concentrations can vary over time due to dose adaptations, for example. The aim of this study was to compare joint models to conventional exposure–response analyses methods with the application of alectinib as proof-of-concept.

**Methods:**

Joint models combine longitudinal pharmacokinetic data and progression-free survival data to infer the dependency and association between the two datatypes. The results from the best joint model and the standard and time-dependent cox proportional hazards models were compared. To normalize the data, alectinib trough concentrations were normalized using a sigmoidal transformation to transformed trough concentrations (TTC) before entering the models.

**Results:**

No statistically significant exposure–response relationship was observed in the different Cox models. In contrast, the joint model with the current value of TTC in combination with the average TTC over time did show an exposure–response relationship for alectinib. A one unit increase in the average TTC corresponded to an 11% reduction in progression (HR, 0.891; 95% confidence interval, 0.805–0.988).

**Conclusion:**

Joint models are able to give insights in the association structure between plasma trough concentrations and survival outcomes that would otherwise not be possible using Cox models. Therefore, joint models should be used more often in exposure–response analyses of oral targeted anticancer agents.

**Supplementary Information:**

The online version contains supplementary material available at 10.1007/s00280-024-04698-w.

## Introduction

Personalized medicine is defined as tailoring the therapy for each specific patient to optimize treatment response [1]. One of the tools that can be used to personalize cancer treatment with oral targeted therapy is therapeutic drug monitoring (TDM), in which drug plasma concentrations are measured and interpreted to improve treatment by dose adaptations, for example. This is mainly useful for oral targeted therapy exhibiting an exposure–response relationship.

Two exposure–response analyses of alectinib have been performed, which showed inconsistent results, as one of the studies found a positive exposure–response relationship and the other did not [2, 3]. In research that investigates whether oral targeted therapies exhibit an exposure–response relationship, plasma trough concentrations at steady state are often used as the pharmacokinetic variable correlated to clinical outcomes. In these studies, longitudinal plasma trough concentrations are often aggregated into a single mean or median value [3–6]. However, pharmacokinetic measurements can vary over time due to factors such as dose modifications, compliance and drug-drug interactions [7]. Therefore, it is of interest to analyse the variability of plasma trough concentrations over time between and within individuals and to determine how these changes influence clinical outcomes. Especially in the case of metastasized disease, it is biologically plausible that continuous suppression of the signalling pathway is crucial for optimal effectiveness.

One of the approaches that can be used to investigate the influence of varying pharmacokinetic measurements over time on survival outcomes are joint models [8–11]. In joint models, longitudinal data and time-to-event data are combined so that one can infer the dependency and association between the longitudinal plasma trough concentrations and survival outcomes. This approach can better assess the effect of the treatment, in which changes in plasma trough concentrations over time due to dose adaptations can be taken into account, for example [12]. Therefore, the aim of this study was to compare joint models to conventional exposure–response analyses methods with the application of alectinib in patients with non-small-cell lung cancer as proof-of-concept.

## Methods

This study was conducted at the Netherlands Cancer Institute-Antoni van Leeuwenhoek hospital (NKI-AvL), Amsterdam, The Netherlands. Patients were included in this study if they were treated with alectinib, if they started treatment between February 2017 and December 2021, and if pharmacokinetic data were available. At the NKI-AvL, plasma samples of patients receiving alectinib were collected during routine follow-up visits to the outpatient clinic as part of the standard of care. In the majority of the cases, the collected plasma samples could not be considered to be trough concentrations, as this is often not feasible to arrange in clinical practice. Therefore, date and time of the last drug intake and plasma sampling were used to calculate trough concentrations of alectinib using log-linear extrapolation in which a plasma elimination half-life of 32 h was used [13]. Plasma concentrations were measured by validated liquid chromatography with tandem mass spectrometry detection [14].

Patient characteristics and survival outcomes were extracted from the electronic medical records, whereas data on plasma samples were extracted from the laboratory database. The conduct of this study was approved by the Investigational Review Board of the NKI-AvL and the need for written informed consent was waived.

### Longitudinal outcome and survival outcome

Alectinib plasma trough concentrations were transformed to normalize the data as this is one of the assumptions of linear mixed effects models [15]. In addition, exposure–response relationships are usually described by the sigmoid E_max_ model, in which a certain drug exposure corresponds non-linearly to a certain drug effect. In these type of models, the response reaches a plateau above a certain exposure, as one can imagine that an alectinib trough concentration of 1000 ng/mL does not result in double the response compared to an alectinib trough concentration of 500 ng/mL. Alectinib plasma trough concentrations were normalized by transformation into transformed trough concentrations (TTC) using this equation:

$$TTC =\frac{{Ctrough}^{\gamma }}{{EC50}^{\gamma }+ {Ctrough}^{\gamma }}\times 100$$

*Ctrough* is the alectinib trough concentration, *EC50* is the alectinib trough concentration that represents the center of the sigmoid curve, and γ is curve-fitting parameter, describing the steepness of the concentration-effect relationship. The *EC50* was set at 600 ng/mL, which is slightly above the target trough concentration of 435 ng/mL used in other studies [16]. Above 600 ng/mL, the TTC increases less than proportional with the trough concentration compared to trough concentrations under 600 ng/mL. Lastly, γ was empirically fixed to ensure that the resulting TTC approximately follows a normal distribution. The factor 100 in the formula ensures that the TTC takes values between 0 and 100, enhancing the interpretability of the results of the Cox models and joint models. The resulting sigmoid curve describing the relationship between the alectinib trough concentration and TTC is shown in the supplementary materials.

The survival outcome was progression-free survival (PFS), which was defined as the time from treatment initiation until the first signs of disease progression by either radiology or clinical signs, or death by any cause in the absence of progression. PFS was estimated using the Kaplan–Meier method and the median follow-up time was estimated with the reverse Kaplan–Meier method [17].

### Covariates

Variables taking into account were weight, sex, previous number of treatment lines, the use of previous ALK inhibitors (e.g. crizotinib and ceritinib), ECOG performance status and the presence of brain metastases at baseline and alectinib dose at time of plasma sampling. Except for weight, all covariates were used as categorical variables with no order.

### Joint model

Joint models consist of two sub-models that are then joined together: a linear mixed effects model and a Cox proportional hazards model.

The linear mixed effects model was used to fit the longitudinal data, in which covariates at baseline and time of plasma sampling were tested on their association with the TTC of alectinib as longitudinal outcome measurements. Subject level random effects for both the intercept and slope were added to cluster the outcome measurements within subjects together, as the linear mixed effects model assume variability in measurements within subjects to be smaller than variability in measurements between different subjects [15]. A Cox proportional hazards model was used to fit the second sub-model using the time-to-event data. Using this sub-model, the association between baseline covariates and PFS were estimated [12, 18].

Lastly, the joint model was fitted using the two sub-models to estimate the association between the longitudinal TTC of alectinib and PFS. In the joint model, the complete trajectory of the TTC is estimated for each individual patient using the included covariates in the first sub-model and the actual TTC measurements. This trajectory of the TTC is then associated with the hazard, i.e. the risk of experiencing an event at a specific time point, in the second sub-model. Via this hazard, the association between the longitudinal measurements of TTC and PFS were determined.

Joint models with different functional forms were tested [18]. These functional forms describe the association structure between the historic trajectory of the TTC of alectinib and the hazard for progression. The basic association structure is to relate the estimated TTC at the time of the most recent measurement, directly to PFS, in which all historic TTCs are used to estimate the current TTC. Using other functional forms, it is possible to associate the average TTC of alectinib, which is the area under the historical TTC trajectory divided by the time, with PFS. In addition, it is possible to combine different association structures together in one joint model. In this study, joint models with the current value, the average exposure and the combination of these two functional forms were tested. In case functional forms are combined, separate hazard ratios are estimated for each functional form. Another functional form is the time-dependent slope of the TTC of alectinib, i.e. how fast the TTC decreases or increases at the time of most recent measurement. Joint models using the time-dependent slope as association structure were not performed in this study as this approach does not reflect the mechanism of action of oral targeted anticancer agents and is more suited for biomarkers. The Watanabe-Akaike information criterion (WAIC) was used to select the best model, in which smaller values are preferred as this indicates better models. Joint models were fitted using the *JMbayes2* package in R version 4.3.1 (R Foundation for Statistical Computing, Vienna, Austria). In order to assess the dependence of our results on the precise form of the sigmoid curve used to transform the trough concentrations, a sensitivity analysis was performed for the joint model with the best association structure, in which the EC50 was set at 500 and 700 ng/mL and the γ was set at 2.

### Cox proportional hazards models

In addition to the joint models, basic Cox proportional hazards models were fitted on the same dataset using the median TTC for each patient as a numerical variable and as a categorical variable. In the model with TTC as a categorical variable, patients were divided into two groups describing whether the median exposure of each patient was adequate or inadequate (reference group) based on the target trough concentration of 435 ng/mL. Similarly, the corresponding time-dependent Cox proportional hazards models were also fitted, as this is the traditional approach to study the association between repeated measurements and the occurrence of an event over time. For the time-dependent Cox proportional hazards models, the time-dependent variable was assumed constant in the time period after the measurement, i.e. the last value was carried forward. The backward elimination procedure was used to determine which covariates are kept in the Cox proportional hazards models. A *p*-value <0.05 was considered statistically significant.

## Results

**Fig. 1:**
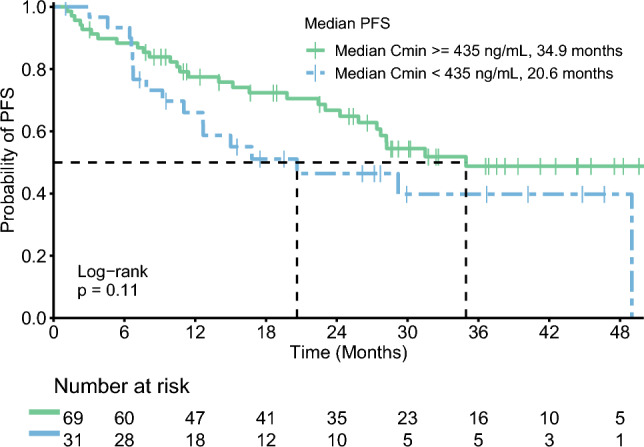
Kaplan–Meier curve for progression-free survival (PFS)

A total of 100 patients were included with 569 repeated measurements. The median follow-up time was 32.4 months (interquartile range of 22.6–44.3 months) and at the time of data cut off, 46 patients had progressed on alectinib treatment. The median number of alectinib plasma samples per patient was 5, with a range from 1 to 17 measurements. Patient characteristics are depicted in Table 1. The longitudinal trajectories of the alectinib trough concentrations are depicted in the supplementary materials. The Kaplan–Meier curves for patients with adequate (≥435 ng/mL) and inadequate (<435 ng/mL) median exposure are depicted in Fig. 1. Median PFS for patients with adequate and inadequate median exposure were 34.9 months (95% confidence interval (CI), 27.4–NA) and 20.6 months (95% CI, 12.7–NA), respectively.

**Table 1:** Patient characteristics

	Patients N = 100
Age, years	
Mean (SD)	58 (13)
Male sex	47
ECOG PS at start treatment	
0	41
1	49
2	7
3	3
Number of previous treatment lines	
0	57
1	27
2	10
3	6
Prior ALK TKI use	
Yes	40
No	60

*ALK TKI* anaplastic lymphoma kinase tyrosine kinase inhibitor (e.g. crizotinib and ceritinib), *ECOG PS* Eastern Cooperative Oncology Group performance status, *SD* standard deviation

The results of the different Cox models and joint models are presented in Table 2. The full results from the different joint models are shown in the supplementary materials. All models were adjusted for prior ALK inhibitor use only as including more baseline covariates did not significantly improve the Cox models. In the basic Cox proportional hazards model, adequate median exposure seemed to be associated with a decreased risk of progression, with a hazard ratio (HR) of 0.645, although not statistically significant. Median TTC as a continuous variable in the basic Cox proportional hazards model did not seem to be associated with a decreased risk of progression. Similar results were observed for the time-dependent Cox proportional hazards models.

**Table 2:** Hazard ratios and 95% confidence intervals (CI) for median transformed trough concentration (TTC) as categorical* and continuous variable for the standard Cox model, current TTC as categorical and continuous variable for the time-dependent Cox model and the joint model with different association structures

	HR	95% CI	*P* value	WAIC	LPML
Cox proportional hazards model					
Categorical (TTC)+ Prior ALKi use: yes	0.645	0.353–1.180	0.155		
	2.229	1.228–4.045	0.008		
Continuous (TTC)+ Prior ALKi use: yes	0.996	0.973–1.019	0.714		
	2.284	1.260–4.139	0.007		
Cox proportional hazards model with time-dependent variable					
Categorical (TTC)+ Prior ALKi use: yes	0.739	0.417–1.309	0.299		
	2.392	1.338–4.276	0.003		
Continuous (TTC)+ Prior ALKi use: yes	0.995	0.975–1.016	0.666		
	2.432	1.360–4.349	0.002		
Joint models with different association structures					
Current value (TTC)+ Prior ALKi use: yes	0.983	0.954–1.012	0.268	4817.6	−2412.7
	2.322	1.129–4.925	0.021		
Average exposure (TTC)+ Prior ALKi use: yes	0.978	0.946–1.008	0.150	4863.5	−2495.4
	2.338	1.099–5.089	0.028		
Current value (TTC)+ Average exposure (TTC) Prior ALKi use: yes	1.094	0.991–1.202	0.078	4813.3	−2408.5
	0.891	0.805–0.988	0.023		
	2.225	1.046–4.853	0.033		

All models were adjusted for prior ALK tyrosine kinase inhibitor (ALKi) use. *adequate compared to inadequate based on the target trough concentration of 435 ng/mL. *LPML* Log pseudo-marginal likelihood, *WAIC* Watanabe-Akaike information criterion

**Fig. 2:**
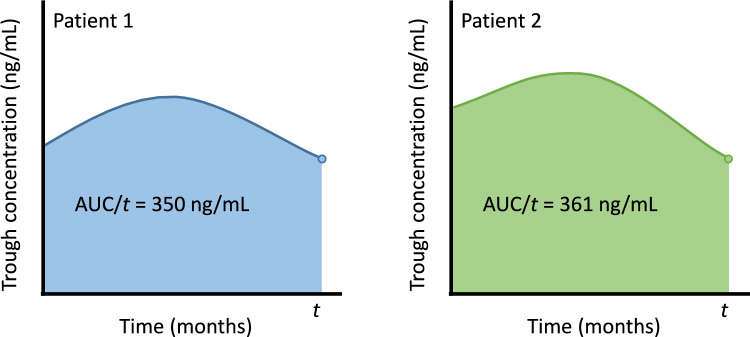
Example for the interpretation of the joint model with current value and average exposure as association structure. Both patients have the same current value (represented by the dot), but a different average exposure. Patient 2 has an 11% reduced risk at progression compared to patient 1 at time *t*. AUC, area under the curve

The final longitudinal sub-model included time and alectinib dose at time of plasma sampling as fixed effects and a subject level random intercept and random slope for time. Previous use of ALK inhibitors was the only variable included in the survival sub-model. The model with both the current value and average exposure was favored as it showed the smallest WAIC value. This joint model found a significant association between the average TTC of alectinib and the risk of progression. A one unit increase in the average TTC corresponded to an 11% reduction in the risk of progression at a given time point (HR, 0.891; 95% CI, 0.805–0.988). Transformed back to trough concentrations, one unit increase in TTC equals the difference between a trough concentration of 350 and 361 ng/mL (Fig. 2) or the difference between a trough concentration of 600 and 617 ng/mL. The results of the sensitivity analyses were consistent with the main analysis (Table S1).

## Discussion

In this proof-of-concept study, we explored whether joint models are better to study exposure–response relationships of alectinib in patients with non-small-cell lung cancer compared to Cox models. Analyzing the same data using joint models and Cox models with or without extensions led to different conclusions. No statistically significant exposure–response relationship was observed in the different Cox models. In contrast, the joint model with the current value in combination with the average TTC of alectinib did show a significant exposure–response relationship.

When comparing the basic Cox model with the time-dependent Cox model, no major differences were observed between the models. In time-dependent Cox models, the last longitudinal measurement is carried forward and assumed to be constant until the new longitudinal measurement. However, this is not representative for pharmacokinetic data that is subjected to biological variation. This possibly explains why the time-dependent Cox models did not perform better than the basic Cox models [10, 12].

In contrast to time-dependent Cox models, joint models are more suitable to use in the analysis of longitudinal pharmacokinetic data and are also able to estimate the complete trajectory of longitudinal measurements. Previous studies have shown that time-dependent Cox models tend to underestimate the true association between longitudinal markers and time-to-event outcomes due to measurement errors and that joint models produce more unbiased estimates [8, 19]. In addition, joint models are able to give insights in the association structure between the longitudinal pharmacokinetic data and time-to-event data as the use of the current value may not always be the best structure to describe the exposure–response relationship, which was also the case in our study [9].

In studies exploring exposure–response relationships of oral targeted anticancer drugs outside trial settings, trough concentrations are the most commonly used pharmacokinetic parameter as obtaining the area under the plasma concentration–time curve in standard clinical practice is less feasible. Previously, Groenland et al*.* observed a prolonged PFS in patients with median trough concentrations above the target trough concentration of 435 ng/mL, whereas no relationship was observed between the trough concentration of alectinib and its active metabolite and overall survival in the study of Morcos et al*.* [2, 3]. The normal multivariable Cox model in our study did not show a statistically significant difference between patients with median trough concentrations above 435 ng/mL compared to patients with median trough concentrations under 435 ng/mL. This is probably explained by the immaturity of the data in our study as alectinib moved from second-line to first-line treatment leading to longer PFS for which a longer follow-up is needed to obtain mature data. In contrast to the Cox models in our study, the joint model with the current value and average exposure showed a significant correlation between an increased average alectinib exposure and longer PFS despite the immaturity of the data. This shows that joint models are able to give insight in the underlying nature of association between pharmacokinetic data and PFS for which conventional methods to study exposure–response relationships are less suited.

Besides the advantages of using joint models, there are also some limitations in our study that need to be pointed out. In this study, we estimated trough concentrations using log-linear extrapolation. Trough concentrations could also be estimated using population pharmacokinetic models, in which interpatient variability is also taken into account. However, as alectinib is dosed twice daily and has a long half-life of 32 h, the relative difference between the peak and trough concentration at steady-state is very small [13]. Therefore, in this specific case, the difference between log-linear extrapolation and population pharmacokinetic-derived exposure metrics will not be relevantly different. In addition, to normalize the longitudinal pharmacokinetic data, trough concentrations were transformed into TTCs using a sigmoid transformation. Although this is a well-known method, it has to be emphasized that the chosen values for the EC50 and the curve-fitting parameter should not be interpreted as the actual relationship between drug exposure and effect as the main goal was to normalize the data. Furthermore, the variability in trough concentrations within patients is assumed to be random in the linear mixed effects sub-model, which is not necessarily the case if patients are nonadherent to their medication, for example. As we did not have data regarding medication adherence or other variables that may contribute to the variability in trough concentrations, these could not be taken into consideration, unfortunately. Data about the moment of alectinib dose adjustments was available, but could not be implemented in the joint model. Ideally, the availability of data on the complete dosing and drug concentration history would result in better estimation of drug effects in which it would also be possible to join non-linear mixed effects models to survival models. Lastly, the joint model with the current value and the average exposure was the best model, in which the model assumes that all pharmacokinetic measurements are of equal importance up to the last measurement time point. This may not be a very reasonable assumption, as recent pharmacokinetic measurements are probably more relevant in regard of survival outcomes. Therefore, it would be of interest to explore this type of association structure, in which pharmacokinetic measurements closer to the event are expected to be of more importance. However, this is not possible yet with the current version of the *JMbayes2* package. In addition to the different association structures of joint models, it is also possible to build a joint model with multiple longitudinal variables. Therefore, this approach may be suitable to study the relationship between drug concentrations, biomarkers and survival outcomes, which in turn can be used to make individual predictions [18].

In conclusion, joint models are able to give insights in the association structure of the pharmacokinetic measurements and survival outcomes that would otherwise not be possible using Cox models. This was also the case in this proof-of-concept study with alectinib, in which an increased average alectinib exposure was correlated with a prolonged PFS even though the data were relatively immature. Therefore, joint models should be used more often in exposure–response analyses of oral targeted anticancer agents.

## Supplementary Information

Below is the link to the electronic supplementary material.

Supplementary file1 (DOCX 691 KB)

## Data Availability

The datasets generated during and analysed during the current study are available from the corresponding author on reasonable request.

## References

[1] Hoeben A, Joosten EAJ, van den Beuken-Van Everdingen MHJ (2021) Personalized medicine: recent progress in cancer therapy. Cancers 13:1–3 10.3390/cancers13020242 PMC7826530 33440729

[2] Morcos PN, Nueesch E, Jaminion F, Guerini E, Hsu JC, Bordogna W et al (2018) Exposure-response analysis of alectinib in crizotinib-resistant ALK-positive non-small cell lung cancer. Cancer Chemother Pharmacol 82:129–138 29748847 10.1007/s00280-018-3597-5 PMC6010493

[3] Groenland SL, Geel DR, Janssen JM, de Vries N, Rosing H, Beijnen JH et al (2021) Exposure-response analyses of anaplastic lymphoma kinase inhibitors crizotinib and alectinib in non-small cell lung cancer patients. Clin Pharmacol Ther 109:394–402 32686074 10.1002/cpt.1989 PMC7891593

[4] Demetri GD, Wang Y, Wehrle E, Racine A, Nikolova Z, Blanke CD et al (2009) Imatinib plasma levels are correlated with clinical benefit in patients with unresectable/metastatic gastrointestinal stromal tumors. J Clin Oncol 27:3141–3147 19451435 10.1200/JCO.2008.20.4818

[5] Carton E, Noe G, Huillard O, Golmard L, Giroux J, Cessot A et al (2017) Relation between plasma trough concentration of abiraterone and prostate-specific antigen response in metastatic castration-resistant prostate cancer patients. Eur J Cancer 72:54–61 28027516 10.1016/j.ejca.2016.11.027

[6] Verheijen RB, Bins S, Mathijssen RHJ, Lolkema MP, Van Doorn L, Schellens JHM et al (2016) Individualized pazopanib dosing: a prospective feasibility study in cancer patients. Clin Cancer Res 22:5738–5746 27470967 10.1158/1078-0432.CCR-16-1255

[7] Groenland SL, Ratain MJ, Chen LS, Gandhi V (2021) The right dose: from phase I to clinical practice. Am Soc Clin Oncol Educ Book 41:92–106 34010057 10.1200/EDBK_319567

[8] Ibrahim JG, Chu H, Chen LM (2010) Basic concepts and methods for joint models of longitudinal and survival data. J Clin Oncol 28:2796–2801 20439643 10.1200/JCO.2009.25.0654 PMC4503792

[9] Mchunu NN, Mwambi HG, Rizopoulos D, Reddy T, Yende-Zuma N (2022) Using joint models to study the association between CD4 count and the risk of death in TB/HIV data. BMC Med Res Methodol 22:295 36401214 10.1186/s12874-022-01775-7 PMC9675185

[10] Baart SJ, Van Der Palen RLF, Putter H, Tsonaka R, Blom NA, Rizopoulos D et al (2021) Joint modeling of longitudinal markers and time-to-event outcomes: an application and tutorial in patients after surgical repair of transposition of the great arteries. Circ Cardiovasc Qual Outcomes 14:E007593 34674542 10.1161/CIRCOUTCOMES.120.007593 PMC8598112

[11] Andersen BL, McElroy JP, Carbone DP, Presley CJ, Smith RM, Shields PG et al (2022) Psychological symptom trajectories and non-small cell lung cancer survival: a joint model analysis. Psychosom Med 84:215–223 34629425 10.1097/PSY.0000000000001027 PMC8831460

[12] Rizopoulos D (2012) Joint models for longitudinal and time-to-event data, with applications in R. CRC Press, Boca Raton, FL

[13] European Medicines Agency Committee for Medicinal Products For Human Use (CHMP) (2023) Alecensa, INN—alectinib. [cited 2023 Sep 23]. https://www.ema.europa.eu/en/documents/product-information/alecensa-epar-product-information_en.pdf

[14] Janssen JM, de Vries N, Venekamp N, Rosing H, Huitema ADR, Beijnen JH (2019) Development and validation of a liquid chromatography-tandem mass spectrometry assay for nine oral anticancer drugs in human plasma. J Pharm Biomed Anal 174:561–566 31255856 10.1016/j.jpba.2019.06.034

[15] Twisk JWR (2019) Applied mixed model analysis. Cambridge University Press, Cambridge

[16] van der Kleij MBA, Guchelaar NAD, Mathijssen RHJ, Versluis J, Huitema ADR, Koolen SLW et al (2023) Therapeutic drug monitoring of kinase inhibitors in oncology. Clin Pharmacokinet 62:1333–1364 37584840 10.1007/s40262-023-01293-9 PMC10519871

[17] Schemper M, Smith TL (1996) A note on quantifying follow-up in studies of failure time. Control Clin Trials 17:343–346 8889347 10.1016/0197-2456(96)00075-x

[18] Rizopoulos D (2023) Predictions from joint models—predictions • JMbayes2 [Internet]. [cited 2023 Oct 18]. https://drizopoulos.github.io/JMbayes2/reference/predict.html

[19] Prentice RL (1982) Covariate measurement errors and parameter estimation in a failure time regression model. Biometrika 69:331–342

