# Supplementary material for: A joint model of longitudinal pharmacokinetic and time-to-event data to study exposure–response relationships: a proof-of-concept study with alectinib
Source: Cancer Chemother Pharmacol. 2024 Jul 11;94(3):453–9. doi: 10.1007/s00280-024-04698-w (PMC11420381; doi:10.1007/s00280-024-04698-w)

**Supplements to manuscript: “A joint model of longitudinal pharmacokinetic and time-to event data to study exposure-response relationships: a proof-of-concept study with alectinib”.**

**Table S1. Sensitivity analyses for the joint model with the current value and average exposure as association structure.** ALKi, ALK tyrosine kinase inhibitor; CI, confidence interval; HR, hazard ratio; JM, joint model; TTC, transformed trough concentration; WAIC, Watanabe-Akaike information criterion.

|  | **HR** | **95% CI** | **P value** | **WAIC** | **P value Shapiro-Wilk test** |
| --- | --- | --- | --- | --- | --- |
| **Joint model 1: EC50 = 600, γ = 1.5** | | | | | |
| Current value(TTC)  Average exposure(TTC)  Prior ALKi use: yes | 1.094  0.891  2.225 | 0.991 – 1.202  0.805 – 0.988  1.046 – 4.853 | 0.078  0.023  0.033 | 4813.3 | 0.102 |
| **Joint model 2: EC50 = 500, γ = 1.5** | | | | | |
| Current value(TTC)  Average exposure(TTC)  Prior ALKi use: yes | 1.106  0.880  2.228 | 1.000 – 1.224  0.782 – 0.978  1.032 – 4.983 | 0.050  0.020  0.043 | 4862.8 | 0.008 |
| **Joint model 3: EC50 = 700, γ = 1.5** | | | | | |
| Current value(TTC)  Average exposure(TTC)  Prior ALKi use: yes | 1.099  0.887  2.227 | 0.982 – 1.246  0.773 – 0.983  1.044 – 4.889 | 0.100  0.047  0.037 | 4775.4 | 0.018 |
| **Joint model 4: EC50 = 600, γ = 2** | | | | | |
| Current value(TTC)  Average exposure(TTC)  Prior ALKi use: yes | 1.080  0.906  2.207 | 1.029 – 1.189  0.814 – 1.012  1.029 – 4.822 | 0.173  0.097  0.042 | 5068.0 | 0.002 |

**Figure S1.** The sigmoid curve describing the relationship between the trough concentration (Ctrough) and the transformed trough concentrations. The two vertical dashed lines represent the target trough concentration of 435 ng/mL and the trough concentration of 600 ng/mL.

**
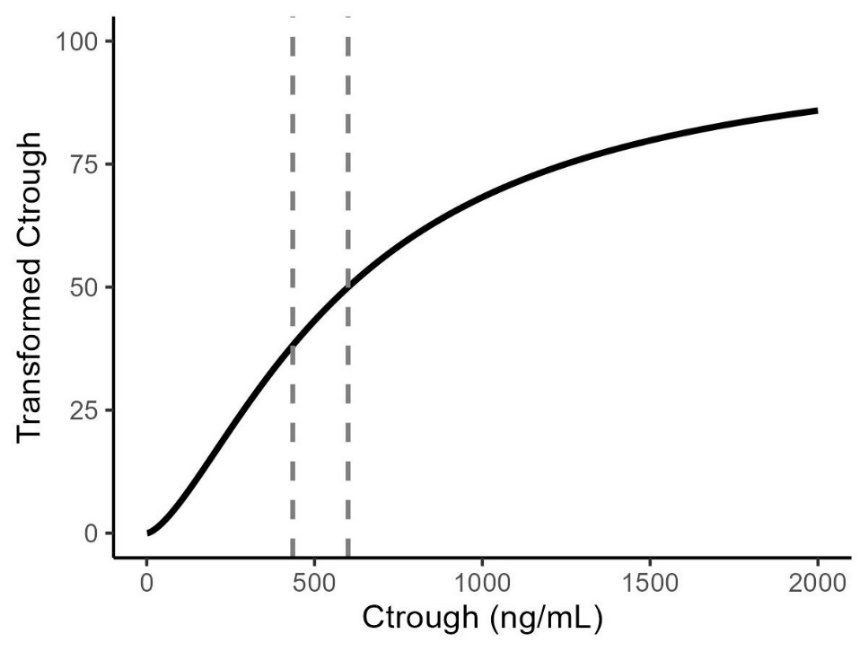
**

**Figure S2.** Spaghetti plots of the individual longitudinal trajectories of a) alectinib trough levels and the b) transformed trough concentrations over time in months. The dotted horizontal line represents the alectinib target trough concentration of 435 ng/mL.

**
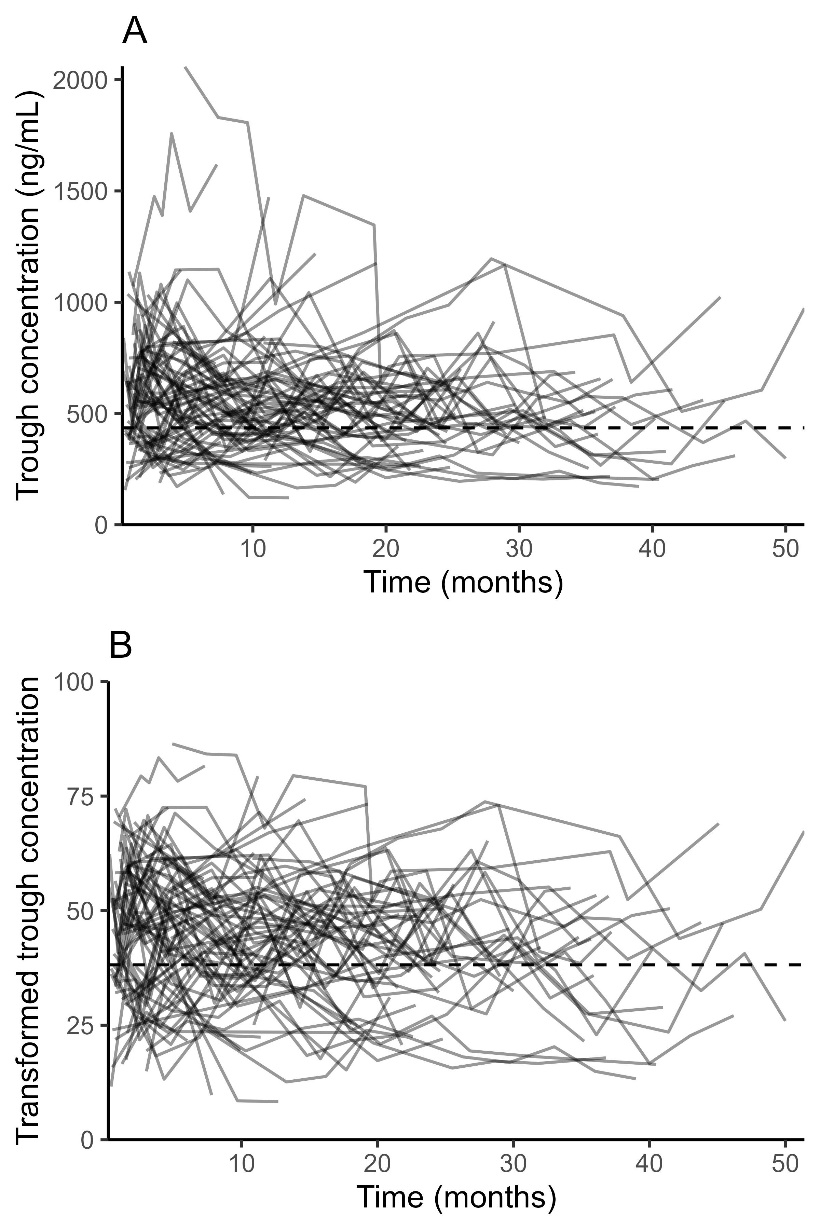
**

**Figure S3. Linear mixed effects sub-model**


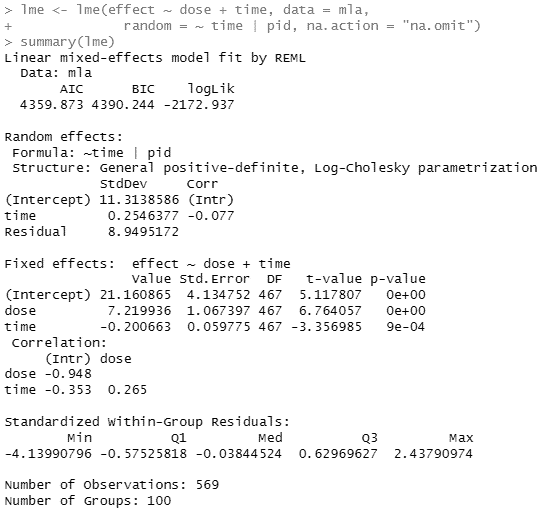


**Figure S4. Time-to-event sub-model**


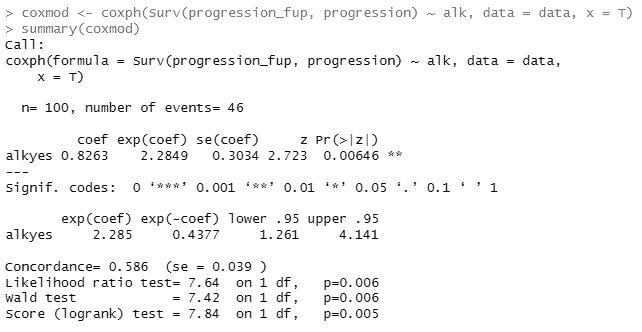


**Figure S5. Joint model with current value as functional form**


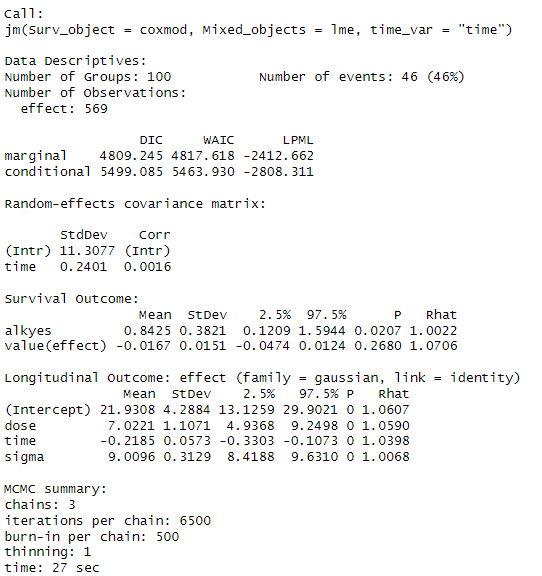


**Figure S6. Joint model with the average exposure as functional form**


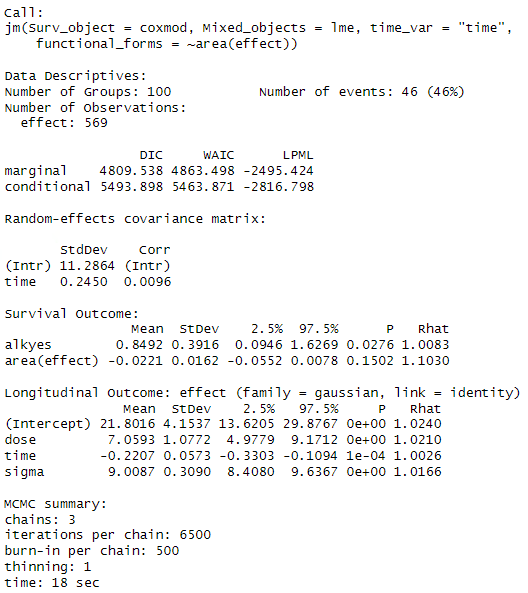


**Figure S7. Joint model with current value and average exposure as functional form**


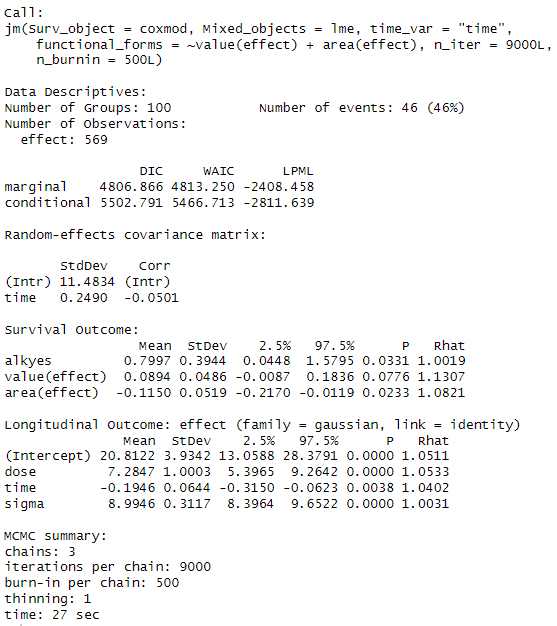

Supplement: Supplementary file 1 — Supplementary file1 (DOCX 691 KB) [file 280_2024_4698_MOESM1_ESM.docx]
